# Biomarkers of Pre-eclampsia in Pregnant Women With Gestational Diabetes and Pre-existing Type 2 Diabetes: A Systematic Review

**DOI:** 10.7759/cureus.53207

**Published:** 2024-01-30

**Authors:** Yasmin Kabir, Norhan Shaykhon, Stephen Atkin

**Affiliations:** 1 Medicine and Surgery, Royal College of Surgeons in Ireland, Manama, BHR

**Keywords:** serum biomarkers, diagnostic markers, maternal morbidity, diabetes mellitus 2, gestational diabetes mellitus, pregnancy, hypertensive disorders of pregnancy, preeclampsia

## Abstract

Pre-eclampsia (PE) is one of the leading causes of maternal and perinatal health morbidity, producing more than 4.6% of complications in pregnancy worldwide. This systematic review was conducted to determine the significance of specific biomarkers in predicting PE in gestational diabetes mellitus (GDM) and type 2 diabetes mellitus (DM). The review measured and explained the significant abnormalities in lipids, blood glucose, cytokines, inflammatory markers, placental proteins, urinary proteins, and other serum biomarkers that contribute to the development of PE in GDM and type 2 DM populations. We searched CINAHL, EMBASE, Medline, Maternity and Infant care, Scopus, and Web of Science. Studies were included if they had a measurable component in the blood serum or urine of women who developed PE and suffered from GDM or pre-existing type 2 DM. A narrative synthesis was conducted instead of a meta-analysis due to the high heterogeneity of data from the studies. A total of 2,593 studies were screened, producing eight relevant studies. Twenty-seven different biomarkers were investigated from the study group of 40 to 1,344 participants. No single biomarker was identified; however, there is a need for further research on specific biomarkers of PE, especially in CRP, FABP4, and microalbuminuria in the GDM-PE group and calprotectin in the type 2 DM population. Many biomarkers were identified as practical in predicting PE when combined with other biomarkers and more data are required to verify the predictability of the diagnostic markers in pregnant women.

## Introduction and background

Pre-eclampsia (PE) is a hypertensive disorder present after 20 weeks of gestation and complicates pregnancy approximately 2-8% globally [1]. The pathophysiology is currently uncertain, but the current diagnosis of PE is characterised by high systolic/diastolic blood pressure of ≥ 140/90 mm Hg [2]. PE may be accompanied by proteinuria ≥ 300 mg/24 h or may cause multiple organ dysfunction. Annually, PE accounts for 50,000 maternal deaths and leads to several maternal complications, including cardiovascular disease (CVD) and neonatal complications (e.g., small for gestational age (SGA) infants) [3].

Although there is uncertainty in the aetiology of PE, hypertensive disorder has associated risk factors, which include two diabetic conditions: gestational diabetes mellitus (GDM) and pre-existing type 2 diabetes mellitus (DM). GDM is defined as hyperglycaemia that develops during pregnancy and resolves after birth, whilst type 2 DM is a permanent condition affecting any age and, in this context, before pregnancy. PE has been identified in 10-14% of patients with type 2 DM, approximately resulting in a two-four-fold risk for PE in patients with pre-existing type 2 DM [4,5]. In diagnosing GDM using the "one-step approach" recommended by the American Diabetes Association (ADA), pregnant women were subjected to a 75-g oral glucose tolerance test between weeks 24 and 28 of gestation. The ADA criteria for identifying GDM were satisfied if any of the three glucose values surpassed specific thresholds: fasting blood glucose (FBG) ≥5.1 mmol/L, one-hour post 75-g oral glucose load ≥10.0 mmol/L, and two-hour post 75-g oral glucose load ≥8.5 mmol/L. For GDM, the condition has a prevalence of up to 25% of the population and can even lead to type 2 DM and CVD after pregnancy [6]. The three conditions are known to increase the risk of adverse events for the mother and the newborn.

The diagnostic tests to confirm PE are currently blood pressure measurements and urinalysis for protein at each antenatal visit [7]. There are no known biomarkers that can definitively state the risk of PE singly in the population [8]. The UK National Screening Committee has not identified any practical screening tests for PE.

There are various studies that have investigated biomarkers for PE such as angiogenic factors, anti-angiogenic factors, and placental proteins (e.g., PIGF, sFLT-1, and HbA1c). However, most of them do not focus on any diabetic conditions. There are very few studies that look at GDM-PE or type 2 DM-PE groups, and in the general collection of studies with diabetes and PE, most look at the biomarker measurements in the type 1 DM-PE group. Wotherspoon et al. [9] did a systematic review to predict PE in the type 1 DM group and concluded that there is no identifiable, single biomarker for the target group. Due to the different pathophysiology of the three diabetic conditions, specific biomarkers may be sensitive to different combinations of diseases. Hence, this review aims to identify potential biomarkers for predicting PE in GDM or in the type 2 DM population.

## Review

Methods

Protocol and Registration

The study was registered on PROSPERO (CRD42021278401) on the 4th of October 2021.

Literature Search

We searched CINAHL, EMBASE, Medline, Maternity and Infant Care, Scopus, and Web of Science for relevant literature. These databases were searched using key terms in combination with other techniques. Table 1 summarises the keywords used to search literature in the various databases.

**Table 1:** Syntax used to search electronic databases. #1 AND #2 AND #3

#1	#2	#3
Pre-eclampsia OR Preeclampsia OR Pregnancy induced hypertension	Gestational diabetes OR Type 2 Diabetes Mellitus OR Diabetes Mellitus OR Insulin-dependent diabetes mellitus	Biomarker OR Proteinuria OR albuminuria OR Metabolomic OR Antioxidant

The search was performed on the 29th of August 2021 on all databases. No language restriction was applied. No specific publication date was applied. All the keywords were searched in combination with Boolean operators, Boolean modifiers, truncations, wildcards, field tags, and medical search headings. Table 2 shows formulated search strings.

**Table 2:** Search strings across six databases.

Database	Search Query	Discovered Studies
CINAHL	(((("Biomarkers"[Mesh]) OR (biomarker*)) OR (proteinuria) OR (pregnancy OR blood OR plasma OR carrier) N3 protein* OR metabolomic OR antioxidant AND ((fft[Filter]) AND (humans[Filter]))) AND (("Pre-Eclampsia"[Mesh]) OR pre-eclampsia OR preeclampsia OR pre N2 eclampsia OR "pregnancy-induced hypertension" OR "pregnancy induced hypertension" OR "pregnancy induced" N3 hypertension OR "pregnancy-induced" N3 hypertension OR pregnancy N3 "induced hypertension" AND ((fft[Filter]) AND (humans[Filter])))) AND ((((("Diabetes, Gestational"[Mesh])) OR (gestational N3 diabet*)) OR ("diabetes mellitus type 2")) OR (OR diabet* N3 "type 2" OR pregnancy N3 diabet*) AND ((fft[Filter]) AND (humans[Filter])))	1744
EMBASE	((("PE":ab,ti OR "Pre-Eclampsia":ab,ti OR "preeclampsia”:ab,ti OR "pre N2 eclampsia":ab,ti OR "pregnancy-induced hypertension":ab,ti OR "pregnancy induced hypertension":ab,ti OR "pregnancy induced":ab,ti OR "hypertension":ab,ti OR pregnancy N3 "induced hypertension":ab,ti OR “gestational N3 diabet*”:ab,ti) AND (Diabetes, Gestational:ab,ti OR Diabetes Mellitus, Type 2:ab,ti OR "diabetes mellitus type 2":ab,ti OR diabet* N3 "type 2":ab,ti OR pregnancy N3 diabet*:ab,ti AND (“Biomarkers”:ab,ti OR "biomarker*":ab,ti OR "proteinuria":ab,ti OR "albuminuria":ab,ti OR "pregnancy”:ab,ti OR (blood OR plasma OR carrier):ti,ab OR N3 “protein*":ab,ti "metabolomic" OR "antioxidant")))	355
MEDLINE	(((MeSH HEADING:exp: Pre-Eclampsia) OR (TOPIC: "PE" OR "preeclampsia" OR "pre N2 eclampsia" OR "pregnancy-induced hypertension" OR "pregnancy induced hypertension" OR "pregnancy induced" OR "hypertension" OR pregnancy N3 "induced hypertension" OR gestational N3 diabet*) AND (( MeSH HEADING: Diabetes, Gestational) OR (TOPIC: Diabetes Mellitus, Type 2 OR "diabetes mellitus type 2" OR diabet* N3 "type 2" OR pregnancy N3 diabet* AND (MeSH HEADING:exp: “Biomarkers”) OR TOPIC: "biomarker*" OR "proteinuria" OR "albuminuria" OR "(pregnancy OR blood OR plasma OR carrier) N3 protein*" OR "metabolomic" OR "antioxidant")))	703
Scopus	(((TITLE-ABS-KEY: "PE" OR "Pre-Eclampsia" OR "preeclampsia" OR "pre N2 eclampsia" OR "pregnancy-induced hypertension" OR "pregnancy induced hypertension" OR "pregnancy induced" OR "hypertension" OR pregnancy N3 "induced hypertension" OR gestational N3 diabet*) AND (TITLE-ABS-KEY: Diabetes, Gestational OR Diabetes Mellitus, Type 2 OR "diabetes mellitus type 2" OR diabet* N3 "type 2" OR pregnancy N3 diabet*) AND (TITLE-ABS-KEY: “Biomarkers” OR "biomarker*" OR "proteinuria" OR "albuminuria" OR "(pregnancy OR blood OR plasma OR carrier) N3 protein*" OR "metabolomic" OR "antioxidant")))	401
Web of Science	(((TOPIC: "PE" OR "Pre-Eclampsia" OR "pre N2 eclampsia" OR "pregnancy-induced hypertension" OR "pregnancy induced hypertension" OR "pregnancy induced" OR "hypertension" OR "pregnancy" OR "induced hypertension" OR gestational N3 diabet*) AND (TOPIC: Diabetes Mellitus, Type 2 OR "diabetes mellitus type 2" OR diabet* N3 "type 2" OR pregnancy N3 diabet* AND (TOPIC: “Biomarkers”) OR TOPIC: "biomarker*" OR "proteinuria" OR "albuminuria" OR "(pregnancy OR blood OR plasma OR carrier) N3 protein*" OR "metabolomic" OR "antioxidant")))	372
TOTAL		3575

Eligibility Criteria

Studies were selected using a pre-defined search strategy across six databases. The duplicates were removed using Covidence. Two reviewers (YK & NS) conducted the screening process independently. Title/abstract screening was based on the relevance of the review topic, which led appropriate studies to undergo full-text screening. The studies were included in the review to meet the inclusion criteria. If there was any conflict in the decision, the two reviewers would follow up with a discussion to make a joint agreement on whether to include a study for the subsequent stage.

Inclusion criteria consisted of participants developing PE with pre-existing type 2 DM, GDM, or both, compared to the control group of not developing PE.

Exclusion criteria were intervention studies, animal studies, genetic studies, case reports, male, newborn/infants, type 1 DM, and eclampsia. Type 1 DM studies were only included if the study also observed participants with type 2 DM or GDM. Additionally, grey literature (research reports, conference proceedings, working papers, etc.) was mostly found on EMBASE and was excluded. 

Data Extraction

Data extraction was performed by two reviewers (YK & NS) independently and determined the final list of results. General study details were extracted: author names, publication year, country of conduct, study design, setting, study period, and DOI. Patient characteristics, such as the number of participants within each study group, age, and trimester measurement, were extracted. 

The outcome of the studies, the definition of PE, the number of participants that have developed PE, and the investigative biomarker to diagnose PE in GDM and/or type 2 DM were taken.

Any clinical measurements that were used to determine the efficacy of the biomarkers were extracted as well: specificity and sensitivity, mean and standard deviation or median and range (including units, if conversion is needed), p-value, results/conclusion formed on the specified biomarker, and any other information about the relation between the specified biomarkers and other tested clinical parameters.

Quality Assessment

**Figure 1:**
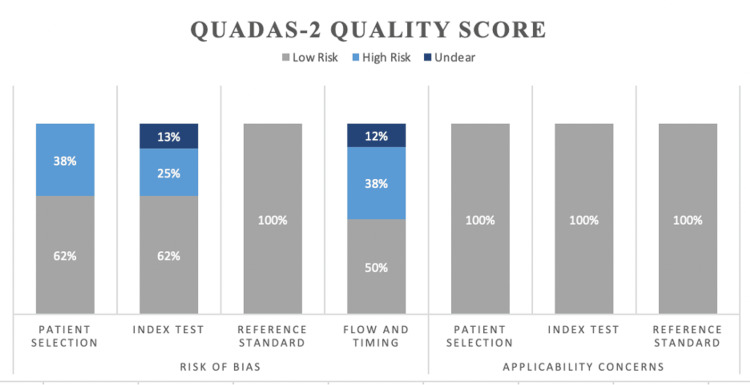
Quality assessment of the eight included studies using the QUADAS-2 tool. Each study was assessed on the risk of bias and applicability regarding patient selection, index test, reference standard, and flow and timing. In terms of patient selection (62% low risk), index test (62% low risk), and flow and timing (50% low risk), there was an overall low risk of bias, especially with the reference standard (100% low risk). The consensus of the applicability of the studies has shown results having a low risk of bias (100%), covering patient selection, index tests, and reference standards.

YK and NS assessed the internal validity of each study after the data extraction. The QUADAS-2 tool template was used to assess the patient selection, the index test, the reference standard, and the flow and timing of each study [10]. If there were any disagreements, a discussion would take place to reach a consensus (Figure 1).

Data Synthesis

The review looked for any differences in the outcome between the GDM or type 2 DM study groups that have developed PE compared to healthy pregnancy or GDM/type 2 DM study groups that have not developed PE. Outcome measures could be the mean biomarker levels or specificity and sensitivity. The studies have been divided by trimester to allow comparisons to be made.

Results

Study Selection

**Figure 2:**
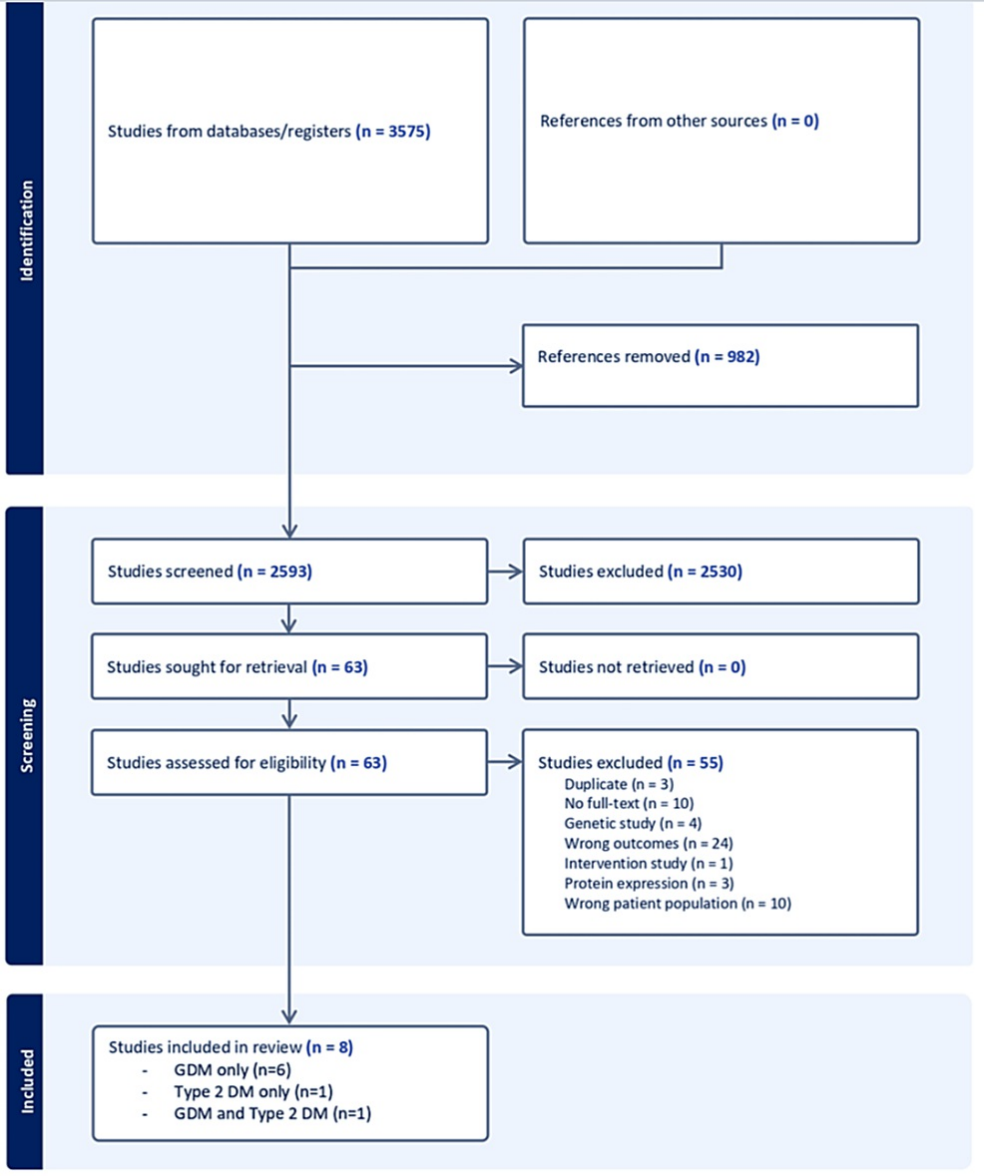
Summarisation of the screening process in the PRISMA flow diagram.

A total of 3,575 records were identified from the search strategy (Figure 2). Once the duplicates were removed, 2,593 records remained. After removing irrelevant records by title and abstract screening, 63 records were selected for the full-text review.

A total of 54 studies were excluded from the review for various reasons, and 24 studies were excluded for wrong outcomes, where most of them had grouped data of two or more diabetic conditions in the development of PE at the time of biomarker measurement (e.g., DM-PE group where DM includes both type 1 and type 2 DM). Due to the significance of the biomarker results in some studies, they will be briefly summarised in the discussion. Another 10 studies were eliminated for assessing the wrong patient population; they did not have GDM, type 2 DM, or a study group that did not develop PE. Genetic studies were not included in the review due to needing a different methodical approach, and, thus, four studies were excluded.

Three studies were found as duplicates that had failed to be detected via Covidence. Another three studies were excluded for measuring protein expression levels rather than measurement of protein levels. One intervention study was also excluded. Of the remaining 19 studies, 11 had no full-texts available online and were excluded. Eight articles were included in the narrative synthesis.

Table 3 outlines the characteristics of the eight selected studies [11-18] dated from 2004 to 2020. The eight studies pertain to data from five cohorts. Recruit of participants occurred across various countries: two studies from China [12,17]; two studies from Australia [11,13]; one study from Norway [15]; one study from Russia [18]; one study from the Czech Republic [16]; and one study from the US [14]. The study size ranged from 40 to 1,344 participants. The percentage of women who developed PE ranged from 2% to 48%. Most of the study designs were cohort except for one cross-sectional study [15] and two case-control studies [12,17].

**Table 3:** Study characteristics of the included studies. DPE includes type 1 DM, type 2 DM, and GDM with PE. Sugulle et al. [3] grouped three diabetic conditions together as the risk of development of PE was similar in all groups. DM = Diabetes mellitus; GDM = Gestational diabetes mellitus; HDL= High-density lipoprotein; LDL= Low-density lipoprotein; VLDL= Very low-density lipoprotein; HbA1c = Glycated haemoglobin; IL = Interleukin; UACR = Urinary albumin to creatinine ratio; CRP = C-reactive protein; hsCRP = High-sensitivity C-reactive protein; TNF-a = Tumour necrosis factor-alpha; PAPP-A = Pregnancy-associated plasma protein-A; fb-hCG = Free beta subunit of human chorionic gonadotropin; FABP4 = Fatty acid binding protein-4; Urinary 2,3d-6k-PGF1 = Urinary 2,3d-6k-Prostaglandin F1; SSD = Statistically significant difference ↑ = p<0.05.1; ↑↑ = p<0.001

	Paper (author, year published)	Total number in study	Number developed pre-eclampsia, n (%)	Biomarker (s) measured	Gestational age (weeks) when biomarker measured	Results/conclusion
GDM	Barden et al. (2004)	184 participants with GDM	22 (12%) in total	Uric acid, Albumin, Creatinine, Urinary microalbumin, Plasma endothelin-1, Urinary 2,3d-6k-PGF1, Total cholesterol, HDL LDL Triglycerides, HbA1c, CRP	31.1 weeks ± 1.5	↑↑Plasma uric acid ↑Urinary microalbumin ↑ CRP
	Cao et al. (2018)	129 (33 participants with GDM)	63 (48%) in total 30 (23%) in GDM + Pre-eclampsia	Triglyceride, HDL, LDL, VLDL, Total cholesterol, IL-17, IL-35, CRP, Fasting blood glucose	PE = 36.47 weeks ± 1.60, DPE = 38.10 weeks ± 1.20	↑↑ Fasting blood glucose ↑ Triglycerides ↑↑ Total cholesterol ↑↑ LDL ↑↑ VLDL ↓↓ HDL ↑↑ IL-17 ↑↑ CRP ↓ IL-35
	Li et al. (2018)	1344 ( 748 participants with GDM)	41 (3%) in GDM	UACR	27 weeks	↑↑ UACR
	Wong et al. (2014)	1015 participants with GDM	22 (2%) in total	FABP4, Total cholesterol, Triglyceride, HDL LDL, HbA1c	24-32 weeks	↑ FABP4 ↑ Triglyceride
	Montoro et al (2005)	150 participants with GDM	29 (19%) in total	Free fatty acids	31.3 weeks ± 2.5	No SSD
	Zak et al. (2019)	40 participants with GDM	3 (7.5%) in total	TNF-a, IL-6, IL-10	2^nd^ trimester 3^rd^ trimester	↑ TNF-a
Type 2 DM	Kapustin et al. (2020)	175 (50 participants with Type 2 DM)	72 (41%) in total 18 (10%) in Type 2 DM	PAPP-A, fb-hCG	11-13 weeks ± 6.0	No SSD
GDM & Type 2 DM	Sugulle et al. (2011)	138 (participants include 11 in Type 2 DM, 63 in GDM and 11 in DPE)	11 (20%) in total	Calprotectin, hsCRP	3^rd^ trimester	↑↑ Calprotectin

Study Characteristics

Over 75% of the studies measured biomarkers across the third trimester (Table 4). Meanwhile, 12.5% measured biomarkers across the second trimester [17], and 12.5% measured biomarkers across the first trimester [18]. The selected studies investigated several biomarkers for predicting PE in patients with GDM or type 2 DM. Two studies measured different cytokines: IL-17 and IL-35 in addition to maternal lipids [12] and IL-6 and IL-10 in addition to tumour necrosis factor-alpha (TNF-a) and haemoglobin A1c (HbA1c) [16]. Other studies focused on different biomarkers, including, free fatty acids (FFAs) [14], calprotectin and high-sensitivity C-reactive protein (hsCRP) [15], pregnancy-associated plasma protein-A (PAPP-A), and free-beta human chorionic gonadotropin (fb-hCG) [18], and urine albumin-creatinine ratio (UACR) [13]. Other studies investigated several biomarkers at once [11,17].

**Table 4:** Biomarkers’ results in the GDM or type 2 DM group for the prediction of PE. p<0.05.^1^ p<0.001.^2^ Total cholesterol.^3 ^High density lipoprotein.^4^ Low density lipoprotein.^5^ Triglycerides.^6^ Hemoglobin A1c.^7^ C-reactive protein.^8^ Very low-density lipoprotein.^9^ Fasting blood glucose.^10^ GDM with PE.^11^ Urine albumin-creatinine ratio.^12^ Adipocyte protein-2.^13^ Free fatty acids.^14^ No statistically significant difference.^15^ Tumor necrosis factor-alpha.^16 ^Pregnancy associated plasma protein-A.^17^ Free beta subunit of human chorionic gonadotropin.^18 ^High-sensitivy CRP.^19^

	Study	Biomarker (s) measured	Gestational age (weeks) when biomarker measured	Results/conclusion
GDM	Barden et al. (2004) [11]	Uric acid, Albumin, Creatinine, Urinary microalbumin, Plasma endothelin-1, Urinary 2,3-dinor-6-keto-prostaglandin-F1, TC^3^, HDL^4^, LDL^5^, TG^6^, HbA1c^7^, CRP^8^	31.1 weeks ± 1.5	↑↑^2 ^Plasma uric acid ↑^1^ Urinary microalbumin ↑ CRP
	Cao et al. (2018) [12]	TG, HDL, LDL, VLDL^9^, TC, Interleukin-17, Interleukin-35, CRP, FBG^10^	PE = 36.47 weeks ± 1.60 DPE^11^ = 38.10 weeks ± 1.20	↑↑ FBG ↑ TG ↑↑ TC ↑↑ LDL ↑↑ VLDL ↓↓ HDL ↑↑ IL-17 ↑↑ CRP ↓ IL-35
	Wong et al. (2014) [13]	uACR^12^	27 weeks	↑↑ UACR
	Li et al. (2018) [17]	FABP4^13^, TG, TC, HDL, LDL, HbA1c	24-32 weeks	↑ FABP4 ↑ TG
	Montoro et al. (2005)[14]	FFA^14^	31.3 weeks ± 2.5	No SSD^15^
	Zak et al. (2019) [16]	TNF-a^16^, Interkleukin-6, Interleukin-10	2^nd^ trimester 3^rd^ trimester	↑ TNF-a
Type 2 DM	Kapustin et al. (2020) [18]	PAPP-A^17^, fb-hCG^18^	11-13 weeks ± 6.0	No SSD
Type 2 DM & GDM	Sugulle et al. (2011) [15]	Calprotectin, hsCRP^19^	3^rd^ trimester	↑↑ Calprotectin

We have found five studies [11-14,16,17] focused on the GDM population only. One study [18] only examined the type 2 DM population, and one study [15] looked at both diabetic conditions. None of the studies reported any predictive measures (e.g., sensitivity/specificity, but all studies reported the statistical significance of the biomarkers that we used for comparison (Table 3).

Lipids were examined in four studies [11,12,14,17] that measured lipids during the third trimester of pregnancy and found that serum levels of total cholesterol (TC), HDL, LDL, and triglycerides (TG) in GDM-PE were not that different from GDM control. This was explained to be due to PE being a late event. Cao et al. [12] measured lipids during the third trimester (36 weeks for PE and 38 weeks for GDM-PE) and found significantly higher levels of serum TG, TC, LDL, and VLDL in DPE in comparison with other populations. Li et al. [17] measured lipids during the third trimester and found no significant difference in HDL, LDL, and TC between the GDM and GDM-PE groups. However, the TG level was higher in the GDM-PE group compared with the GDM group. Montoro et al. [14] measured FFAs during the third trimester (31 weeks) and found no significant difference between pre-eclamptic and non-pre-eclamptic women with GDM.

Blood glucose was examined in four studies [11,12,16,17]. Barden et al. [11] examined the level of blood glucose during the third trimester using HbA1c, which was found elevated in patients with GDM-PE, but not significantly; it had significantly higher insulin levels and a higher HOMA score than GDM-N. Cao et al. [12] measured the level of fasting blood glucose (FBG) during the third trimester in patients with GDM, PE, DPE, and normal pregnancy (NP); elevated levels of FBG were seen in DPE and GDM compared with PE. Li et al. [17] measured the level of FBG and HbA1c between GDM-GH/PE and GDM group without PE during the third trimester, all displaying no significant differences. Zak et al. [16] measured the level of HbA1c during the third trimester.

Cytokines were examined in two studies [12,16] that measured the production of TH17-related cytokines (e.g., IL-17 and IL-35) during the third trimester. Results displayed significantly higher serum levels in patients with GDM, PE, and DPE than those with NP, while it was higher in DPE and PE than in those with GDM. Lower levels of IL-35 were in patients with GDM, PE, and DPE compared to those with NP, especially in those with DPE and PE compared to those with GDM.

Zak et al. [16] measured the production of TNF-a, IL-10, and IL-6 during the second and third trimesters, which was divided into ‘low’ and ‘high’ values. A significant difference was found in TNF-a with no effect in IL-10 and IL-6.

Three studies measured the level of inflammatory markers ([11,12,15]). Barden et al. [11] measured the level of CRP in women with GDM-PE compared to women with GDM-N during the third trimester, which was found significantly elevated in GDM-PE compared to GDM-N. Cao et al. [12] measured the level of CRP in women with GDM, PE, DPE, and NP, where CRP was significantly elevated within the GDM, PE, and DPE groups. Sugulle et al. [15] measured the level of calprotectin and hsCRP in diabetics with PE (DPE) and the control group during the third trimester. Calprotectin concentration in the serum was significantly elevated in patients with GDM, type 1 DM, and type 2 DM compared to the control group. hsCRP concentration in the serum had no significant difference in the DPE group compared to that in the control.

One study [18] measured the level of placental proteins PAPP-A and fb- hCG during the first trimester in patients with TDM1, TDM2, and the control group.

The median level of PAPP-A was significantly lower in patients with T1DM and T2DM than in the control group. There were no significant differences in fb-hCG between the diabetic and control groups.

Two studies measured the level of urinary proteins [11,13]. Barden et al. [11] measured the difference in urinary microalbumin and urinary 2,3-dinor-6-keto-PGF1 between GDM patients with PE and without PE during the third trimester. Urinary microalbumin was significantly elevated at the diagnosis of GDM-PE patients compared to GDM. However, there was no significant difference in 2,3-dinor-6-keto-PGF1 between both groups.

Wong et al. [13] measured the difference in urine albumin-creatinine ratio during the second and third trimesters in women with GDM, and there was no significant difference in UACR levels.

One study assessed many other biomarkers [11]. This includes uric acid, albumin, creatinine, and plasma endothelin-1 levels during the third trimester in patients with GDM-PE and GDM-N. Urinary microalbumin and serum uric acid were elevated during GDM diagnosis, but serum creatinine and endothelial levels showed no significant difference between the two groups.

Discussion

The review identified 27 different biomarkers in eight studies to predict PE. In the GDM population, 25 biomarkers were assessed in seven studies compared to two studies of the type 2 DM population with four biomarkers. Out of the seven studies, only five studies [11-13,16,17] of the GDM population were found statistically significant in at least one of the biomarkers. None of the studies was statistically significant for the type 2 DM population. The data extracted varied in terms of outcome interests, which impeded a meta-analysis from being performed. Additionally, due to the high heterogeneity of the data, a meta-analysis could still not be performed. The review showed limited evidence where a single biomarker cannot be chosen as a diagnostic tool in the target population to predict PE. Most of the studies involved using multiple biomarkers and the patient's clinical history to diagnose PE. Even fewer studies examine biomarkers in the type 2 DM population only; some of the excluded studies looked at biomarker measurement in type 1 DM and type 2 DM together rather than separately, which may introduce inaccuracy of data interpretation. Although one study [15] had stated that the probability of the development of PE in type 1 DM, type 2 DM, and GDM were all similar and hence, grouped the results to represent all three conditions; other studies with grouped data show to be unclear if the likeliness to develop PE in more than one diabetic condition is similar.

Gestational Diabetes Mellitus

Three studies [11,12,17] measured four lipid biomarkers, namely, TC, TG, HDL, and LDL, to predict PE in the GDM group and have reported different results. Whilst Barden et al. [11] reported no statistically significant difference between GDM with PE and the GDM-control group, a study [12] reported significant results (p<0.01) for the four lipid biomarkers and in VLDL. Lie et al. [17] reported statistical significance (p<0.05) in TG only. The three studies investigated similarly around the beginning of the third trimester (28.20-33.49 weeks). However, the results of Barden et al. [11] could potentially be affected by the development of PE occurring in the late third trimester of pregnancy (37.6 weeks). At different gestational ages, biomarker measurements are reported to increase TG, TC, LDL, and VLDL levels with decreased HDL levels; it may be caused by endothelial damage and vascular dysfunction. After 37 weeks, a study reported that the lipid levels might not be as different from the non-PE group [19]. A similarity between the two studies [2,12] is higher BMI in the PE group than the non-PE group. Lie et al. [17] reported no difference. Overweight pregnant women have an increased risk of PE and are expected to have a threefold increase of GDM compared to women of average weight [20,21]. It is unclear if the lipid biomarkers are specific to the development of PE in the GDM group or groups with high BMI or are only effective as a diagnostic tool at different gestational ages.

HDL levels are usually increased during pregnancy since it is a vasodilator and enables the transport of excess cholesterol to the liver for excretion [22]. Cao et al. [12] reported a decrease in HDL levels, whilst Barden et al. [11] and Li et al. [17] reported no difference. A systematic review for the diagnosis of PE [23] reported elevated HDL levels in pre-eclamptic patients in the third trimester. There is uncertainty if HDL levels can be used as a predictive measure, separately or in combination, as the results vary in the GDM-PE groups; thus, further research is needed.

Li et al. [17] showed no correlation between the four biomarkers and FABP4 in the GDM-PE group, which has been positive in the Kralisch et al.'s [24] study. FABP4 serum levels were significantly higher in the GDM-PE group and may be associated with the thickness of the carotid artery, although the biological nature remains majorly unknown [25]. A study on type 1 DM [9] showed an increased risk of PE with high levels of FABP4 as a single biomarker in the second trimester. Another study [26] reported a similar correlation for the risk of PE with elevated FABP4 serum levels. The majority of the FABP4 results seem to be consistent with the recent studies, and further research should be conducted into the GDM group specifically to identify if FABP4 can be used as a diagnostic tool for PE.

Plasma FFA concentration showed the same difference in the GDM-PE and control groups [14]. A study [27] reported FFA levels to be 67% higher in the PE group compared to the normotensive group and an association between elevated FFAs and increased serum uric acid levels in the PE group. Elevated FFAs may be seen as a complication of vascular dysfunction in PE and are formed from a combination of palmitic, oleic, and linoleic FFAs in the plasma [28]. FFAs may not be suitable as a single biomarker for the diagnosis of PE but can be used as a combination of different FFAs and other biomarkers (e.g., uric acid).

In Cao et al. [12], the study reported elevation of FBG in the GDM-PE (p<0.05) group compared to that of normal pregnant women. However, FBG levels of GDM-PE were the highest out of all groups, reporting p<0.001 compared to that of the PE group, and the GDM group being the second-highest level and reporting p<0.01 compared to that of the PE group. FBG may affect other vessels in the GDM population, which may cause an association between GDM and the development of PE. However, this may be due to different glycaemic controls between the groups [29].

HbA1c levels were studied [11,17] and reported elevation in the biomarker levels in GDM-PE compared to that in control, but not significantly. Ho et al. [30] reported that HbA1c might be associated with adverse events in pregnancy around the second trimester. Cavero et al. [31] concluded that high HbA1c levels could indicate PE in type 1 DM. HbA1c may be a potential biomarker for GDM [32] or type 1 DM- PE, but for the GDM-PE group, it is vastly unclear.

Urinary 2,3-dinor-6-keto-PGF1 alpha was reported to have reduced levels of the biomarker in GDM-PE compared to GDM and normal pregnant women, although not significantly so. The biomarker is a metabolite of prostacyclin and may contribute to endothelial dysfunction at reduced levels, consistent in the Lewis et al.'s [33] study of PE pregnancies. Reduced levels may be associated with increased levels of plasma TG [34]. Currently, few recent studies analysed prostacyclin and its metabolites with PE, and even fewer in diabetics with PE groups.

Microalbuminuria was significantly higher (p<0.05) in the GDM-PE group with a similar association in Wong et al. [13] in UACR (p<0.01). However, serum albumin and creatinine levels were not significantly different from control. Higher albumin excretion has been proven to predict PE in pre-existing diabetes at early pregnancy, with one in two risks of developing PE [35]. UACR has already been used as a diagnostic tool to predict PE and GDM [36] and may be used in either condition to anticipate one another.

Proinflammatory cytokines such as IL-6 have shown elevated levels linked with increased blood pressure [37]. Zak et al. reported no difference in the IL-6 levels between the GDM-PE and GDM groups; however, they negatively correlated with TNF-a and IL-10. Similarly, IL-6 and TNF-a reported no statistically significant difference in the prediction of PE in other studies [16,38,39] and did report a significant, positive correlation between the TNF-a and GDM-PE group in the third trimester. A study has shown that elevated TNF-a levels can cause placental ischaemia [40] as it begins to be present at the early and late stages of pregnancy [41]. A review was conducted on IL-10 to show a decreased cytokine level in early pregnancy for the PE group [42], which becomes inconsistent with Zak et al.'s study [16].

Cao et al. [12] investigated two cytokines, IL-17 and Il-35, and found no difference between the study groups. The role of IL-17 involves forming a complex with T-regulatory cells to induce systemic inflammation [43]. IL-17 may contribute towards vascular and endothelial dysfunction as this study [12] does show a positive correlation of IL-17 with proteinuria, diastolic blood pressure (DBP), and BMI. It may also suggest that IL-17 and IL-35 imbalance can cause endothelial dysfunction, which may be correlated with increased blood pressure and proteinuria [44].

Overall, most cytokines' studies are aimed at a potential PE treatment rather than a diagnostic biomarker. It is unclear whether cytokines can be used in combination or independently, as a few inconsistent studies have been conducted.

Hyperuricaemia has been consistently associated with PE [45] and has produced a significant difference (p<0.01) in the GDM-PE group [11]. High levels of the biomarker may have resulted from insulin resistance. A study [46] found a uric acid ratio of less than 1.5 to be a significant indicator for pregnant women who are not expected to develop PE. The findings are distinguishable from the other biomarkers tested as they could potentially be an independent biomarker for the GDM population for the risk of PE without clinical history. However, some studies show that uric acid levels only increase after PE clinical symptoms have begun [47].

Many studies propose endothelin-1 as an inductor for hypertension in PE [48] but produced no difference in the GDM-PE group [11]. However, several other studies correlated endothelin-1 levels in the PE group to NP, resulting in high levels of a two-three-fold risk for PE [49].

As for CRP, Barden et al. and Cao et al.'s [11,12] studies agree that it may be a biomarker for the prediction of PE in the GDM group; Sugulle et al. [15] reported no significant difference in the DPE group. In another study, CRP correlated significantly with DBP, proteinuria, and uric acid levels in the PE group [50]. A review [51] concluded that CRP levels higher than 7-15 mg/L in the first trimester should have preventative measures in place as their PE risk may increase. Potentially, CRP can become an independent biomarker for PE in the GDM group, specifically for low-risk groups without risk factors or clinical history of PE.

Type 2 Diabetes Mellitus

Only two studies investigated four placental biomarkers in predicting PE in the type 2 DM group. Kapustin et al. [18] reported that PAPP-A and fb-hCG serum levels were insignificant in the type 2 DM- PE group in the first trimester compared to that of the control group. The two biomarkers are standard biomarkers in foetal screening for Down syndrome [52]. Another study [53] reported that low levels of PAPP-A (<10th percentile) were an indicator of a high risk of developing PE, whilst a review concluded PAPP-A and fb-hCG had a low predictability accuracy for PE [54].

Whilst hsCRP reported no significant difference in the DPE group [15], calprotectin had significantly high levels compared to the control group (p<0.01). Calprotectin contributes to endothelial dysfunction and may cause systemic inflammation [55]. Pergialiotis et al. [56] reported a positive correlation between high levels of calprotectin and the risk of PE; however, there is still limited research on the prediction of PE.

Generally, most of the biomarkers investigated in the eight studies show no consistent results or lack of studies that are in accordance with the biomarkers' results. For some, the biomarkers are helpful for the prediction of PE when used in combination with other markers. Nonetheless, there is a strong suggestion for further research in CRP, FABP4, and microalbuminuria as a single biomarker for PE in the GDM population and calprotectin in the type 2 DM population.

Excluded Studies

As mentioned in the results, a few excluded studies had grouped data (e.g., type 1 DM + type 2 DM) and had no separate data available for the development of PE in each condition, even after contacting the authors. This review did not want to duplicate any findings in another study that looked at the prediction of PE in type 1 DM [9]. Thus, after a preliminary investigation of all diabetic conditions with PE, the review narrowed down to GDM and type 2 DM. It is important to note that having grouped data might be inaccurate for each of the diabetic conditions in predicting PE since the conditions' pathophysiology are different from each other. Not many studies look at the probability of each diabetic disease developing PE for each biomarker, except for one included study that stated the probability for PE was similar [3]. This led to the exclusion of studies with grouped data in this review. This review looked briefly at some of the excluded studies that may be significant in their findings (Table 4) but will not affect the review's results.

Strengths and Limitations

The review adhered to a rigorous search strategy across six databases with no language or date restriction. Any outcome measurements were extracted, and after a full-text review, all eight studies had a difference in biomarker measurements in the study groups, which was used for comparison. All eight studies underwent QUADAS quality checks to ensure that biases were assessed. The review covered an extensive range of biomarkers in different cohort studies and introduced several pathways to apply a more focused approach.

The limitation of the review was primarily due to the inclusion of a few relevant studies. All included studies had a wide variety of outcomes, which did not allow a meta-analysis to be performed. Some of the excluded studies could have been included if they did not lack numerical values for the outcome of interest; many authors were contacted, but most were nonrespondent. Some studies did not state the period between the diagnosis of each index and the reference test. The definition of preeclampsia and diagnosis of GDM varied across studies.

Whilst only a few studies were found in the review, the study introduces a need for further research into the biomarkers for predicting PE in GDM and type 2 DM populations.

## Conclusions

This review has demonstrated many potential areas for further research in predicting PE as a single biomarker, specifically for CRP, FABP4 and microalbuminuria in GDM, and calprotectin in the type 2 DM population. Many of the biomarkers investigated in the review have been shown to be more effective in predicting PE combined with other markers. Due to a lack of relevant studies and a small study population, we cannot find an independent biomarker definitively. Hence, a significant number of studies of substantial size is needed to help verify the existing studies and explore other biomarkers that can identify the affected individuals, which would allow preventative treatment to ensue.
